# Genomic and gene expression profiling of minute alterations of chromosome arm 1p in small-cell lung carcinoma cells

**DOI:** 10.1038/sj.bjc.6602452

**Published:** 2005-03-22

**Authors:** L-J Henderson, B P Coe, E H L Lee, L Girard, A F Gazdar, J D Minna, S Lam, C MacAulay, W L Lam

**Affiliations:** 1 British Columbia Cancer Research Centre, 675 West 10th Avenue, Vancouver, BC, Canada V5Z 1L3; 2 Department of Pathology and Laboratory Medicine, University of British Columbia, G227-2211 Wesbrook Mall, Vancouver, BC, Canada V6T 2B5; 3 Hamon Center for Therapeutic Oncology Research, University of Texas Southwestern Medical Center, 6000 Harry Hines Blvd., Dallas, TX 75390, USA

**Keywords:** small-cell lung carcinoma, array CGH, 1p, gene expression

## Abstract

Genetic alterations occurring on human chromosome arm 1p are common in many types of cancer including lung, breast, neuroblastoma, pheochromocytoma, and colorectal. The identification of tumour suppressors and oncogenes on this arm has been limited by the low resolution of current technologies for fine mapping. In order to identify genetic alterations on 1p in small-cell lung carcinoma, we developed a new resource for fine mapping segmental DNA copy number alterations. We have constructed an array of 642 ordered and fingerprint-verified bacterial artificial chromosome clones spanning the 120 megabase (Mb) 1p arm from 1p11.2 to p36.33. The 1p arm of 15 small-cell lung cancer cell lines was analysed at sub-Mb resolution using this arm-specific array. Among the genetic alterations identified, two regions of recurrent amplification emerged. They were detected in at least 45% of the samples: a 580 kb region at 1p34.2–p34.3 and a 270 kb region at 1p11.2. We further defined the potential importance of these genomic amplifications by analysing the RNA expression of the genes in these regions with Affymetrix oligonucleotide arrays and semiquantitative reverse transcriptase–polymerase chain reaction. Our data revealed overexpression of the genes *HEYL*, *HPCAL4*, *BMP8, IPT*, and *RLF*, coinciding with genomic amplification.

Greater than 42 000 new cases of small-cell lung cancer (SCLC) are diagnosed annually in the United States, representing approximately 20% of all new lung cancer cases. Median survival time is 12–36 months and the majority of patients eventually die from the disease (Minna *et al*, 2003). Advancing our understanding of the molecular characteristics of SCLC may lead to the ability to better diagnose and treat the disease. One approach to this is the detection of regions of genetic alteration in the tumour genome in order to identify the genes that are causal to the disease. Identification of novel genetic changes correlated to specific human malignancies has typically been a tedious and laborious process. Recently, high-throughput and high-resolution detection of such alterations has been made possible through the use of bacterial artificial chromosome (BAC) array comparative genomic hybridisation (CGH) (Pinkel *et al*, 1998). This technique has been applied to map alterations across the genome at 1 megabase (Mb) resolution (Snijders *et al*, 2001; Greshock *et al*, 2004), at higher resolution for specific chromosomal regions and chromosome arms in several tumour types (Buckley *et al*, 2002; Garnis *et al*, 2003, 2004a), and, most recently, the whole human genome (Ishkanian *et al*, 2004).

Approximately 120 Mb of DNA span human chromosome arm 1p. Multiple regions on this chromosome arm have been implicated in SCLC and non-small-cell lung cancer (NSCLC) (Girard *et al*, 2000; Nomoto *et al*, 2000; Chizhikov *et al*, 2001; Ashman *et al*, 2002). Here, we describe the construction of a unique resource for analysing 1p. In all, 642 fingerprint-verified, physically mapped, overlapping BAC clones serve as target DNA for high-resolution BAC array CGH. Using this resource, we identified novel regions of DNA alteration in SCLC cells. We further defined the role of these alterations by employing Affymetrix oligonucleotide arrays and semiquantitative reverse transcriptase–polymerase chain reaction (RT–PCR) to analyse gene expression.

## MATERIALS AND METHODS

### BAC array construction and CGH

To design a minimal tiling path, BAC clones from the RPCI-11 library (Osoegawa *et al*, 2001) were selected from the FPC (Fingerprinted Contigs) database (International Human Genome Mapping Consortium, 2001). Wherever possible, sequenced and overlapping clones were chosen. Clones were obtained from glycerol stocks of the RPCI-11 library. DNA isolation and restriction enzyme digestion was performed as described previously (Marra *et al*, 1997). Each clone's identity was verified by comparing its *Hin*dIII DNA fingerprint to that found in the FPC database. The BAC DNA amplification, array printing, hydridisation, and analysis were performed as described previously (Garnis *et al*, 2004a). A log 2 signal ratio of zero represents the most common copy number between the sample and normal reference DNA. The array was subjected to normal/normal hybridisation in order to eliminate clones that deviated from 0 by greater than +0.2 or less than −0.2. Clones with standard deviations among the triplicate spots greater than 0.075 were excluded from further analysis. A gain of copy number was defined as a log 2 ratio of greater than 0.2, and a loss as a log 2 ratio of less than −0.2. The average log 2 ratio of two or more adjacent clones was required to be above the threshold in order for a region of alteration to be defined.

### Cell lines

The lung cancer cell lines used were established at the National Cancer Institute (Phelps *et al*, 1996), with the exception of HCC33, which was generated at the Hamon Center for Therapeutic Oncology Research (Gazdar *et al*, 1998). NHBEC (normal human bronchial epithelial cells) and SAEC (small airway epithelial cells) were obtained from Clonetics (San Diego, CA, USA). HBEC2-KT, HBEC3-KT, HBEC4-KT, and HBEC5-KT are normal human bronchial epithelial cell lines immortalised with CDK4 and htert (Ramirez *et al*, 2004). Culturing conditions and DNA isolation were described previously (Girard *et al*, 2000). RNA isolation was carried out using the RNeasy Midi kit (Qiagen, Valencia, CA, USA), together with in-column DNase1 treatment.

### Gene expression profiling using Affymetrix arrays

RNA fluorescent-labelling reaction and hybridisation were performed using the Affymetrix GeneChips HG-U133A and HG-U133B according to the manufacturer's instructions (http://www.affymetrix.com). The arrays consist of 22 283 (HG-U133A) and 22 645 (HG-U133B) probe sets, which together amount to 24 698 unique genes based on Unigene build 163. Total RNA (5 *μ*g) was used for each reaction. Microarray analysis was performed using Affymetrix MicroArray Suite 5.0 and an in-house Visual Basic software MATRIX 1.24 (Girard *et al*, manuscript in preparation). Briefly, array data were median normalised and samples (or averages of samples) were compared against each other by calculating log ratios for each gene. Specifically, a lower signal threshold was set at 100 to reduce the amount of noise and the log ratio of one signal *vs* another was calculated by dividing the signals and computing the log base 2. These log ratios were calculated only when both signals were Present (i.e. the signal-associated detection *P*-values were less than 0.05) or when the higher signal was Present and the lower signal was Absent (*P*-value more than 0.05).

### Gene expression profiling using semiquantitative RT–PCR

Analysis was conducted as described previously (Garnis *et al*, 2004b). PCR conditions were: one cycle of 95°C, 1 min; amount of cycles as listed for each gene of 95°C, 30 s; 62°C, 30 s; 72°C, 1 min and a 10 min extension at 72°C, except Notch2 where annealing temperature was 55°C. Gene-specic primers were used to assay the quantity of: HEYL (5′agaatccctagtggggctgt3′; 5′gatgcaagtccttgaccaca3′; 35 cycles), HPCAL4 (5′tgctgatggaaggaacaaca3′; 5′tgtgctttgtggcaagtctc3′; 35 cycles), BMP8 (5′ttatctgcgcctccattttc3′; 5′tatgtgccaactctgcttcg3′; 35 cycles), PPIE (5′gaagccaaagcagaaggtga3′; 5′atatcccaaatgctgcttgc3′; 32 cycles), IPT (5′ggagggaggggtatgtttgt3′; 5′gacacatcagccacacaagg3′; 30 cycles), RLF (5′tttgatgattgggagccttc3′; 5′aaaggtgggattgcagtcag3′; 35 cycles), CAP1 (5′catccagggcagttaatgga3′; 5′agcatgacagggaaaaggag3′; 30 cycles), Notch2 (5′ggaatggtggcagaactgat3′; 5′ggcatggtactcttggcact3′; 30 cycles). *β*-actin expression levels were used to normalise samples.

### Statistical analysis of Affymetrix expression data

Only those Affymetrix probe sets that demonstrated a detection score of Present or Marginal (*P*<0.06) in at least 50% of the samples were analysed further. Statistically significant expression changes were identified using a Mann–Whitney *U*-test for those genes that passed the detection criteria. For determination of expression differences between the SCLC and normal cell lines, a two-tailed test was utilised and the direction of change noted. In determining a correlation between overexpression and genomic amplification, a one-tailed test was used. Genes were considered to exhibit aberrant expression if the *P*-value crossed a threshold of 0.05.

### Statistical analysis of RT–PCR expression data

A ratio was obtained for each sample by dividing the intensity of the gel band for the gene of interest by the corresponding *β*-actin band. These ratios were then compared, using the Mann–Whitney *U*-test as described above, between samples exhibiting genomic amplification and samples exhibiting retention in the two regions of interest. If the *P*-value for the comparison was less than 0.05, and the data were consistent with the Affymetrix analysis, the gene was considered to have different expression between the two groups.

## RESULTS AND DISCUSSION

The minimal tiling path for the 1p array consists of 642 BAC clones, of which 373 (58%) are sequenced clones, covering from 1p11.2 to 1p36.33. FPC clone order was compared to the golden path assembly on the July 2003 version of the UCSC Human Genome Browser (http://genome.ucsc.edu) (Kent *et al*, 2002) using the sequence accession number, BAC end sequence, or existing FISH maps. In total, 438 (68%) clones are confirmed with at least one of these methods and the remaining 204 BACs are ordered by their FPC location. The complete clone list has been made publicly available at http://www.bccrc.ca/cg/ArrayCG H_Group.html.

**Figure 1:**
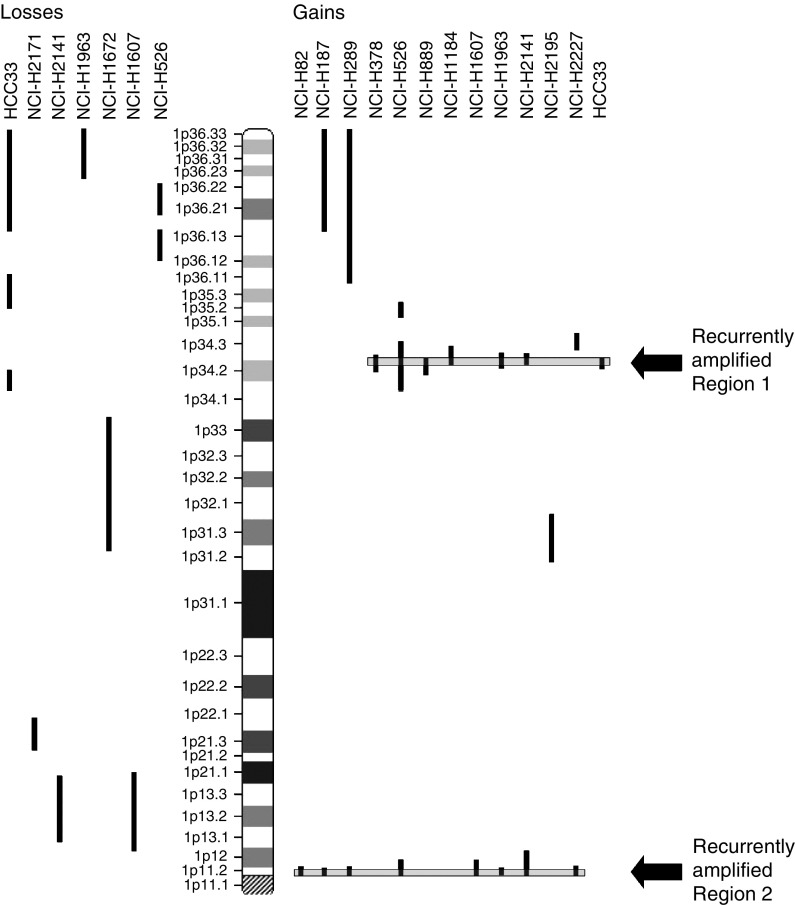
Alignment of 1p array CGH profiles from 15 SCLC cell lines. Gains are shown to the right of the chromosome arm and losses to the left. Two recurrent amplifications are revealed, one at 1p34.2–p34.3 and the second at 1p11.2.

**Figure 2:**
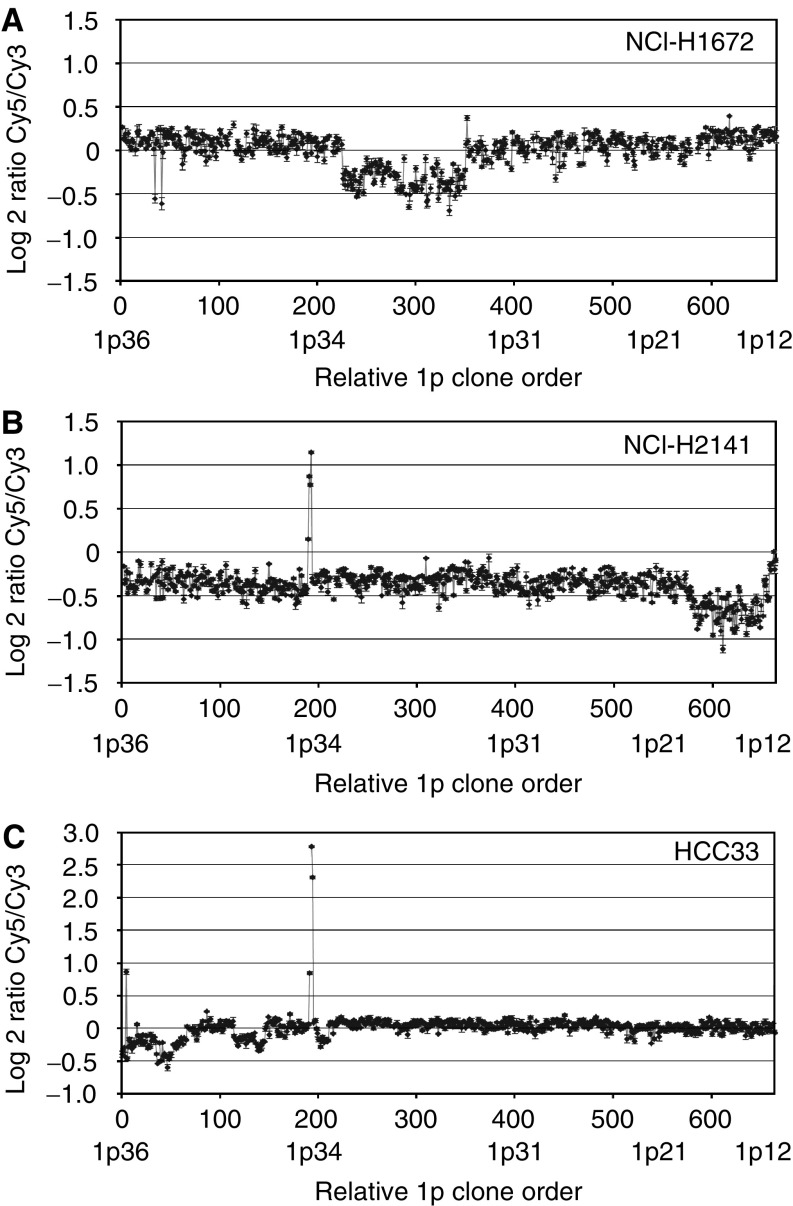
Array CGH profiles of SCLC cell lines. (**A**) Profile of NCI-H1672 demonstrating a large deletion at 1p31.2–p34.1. (**B**) Profile of NCI-H2141 demonstrating a small amplification at 1p34.3, deletion at 1p13.1–p13.3, and amplification at 1p11.2–p12. (**C**) Profile of HCC33 showing a small amplification at 1p34.2–p34.3 and multiple distinct deletions from 1p34.1 to p36.33.

We chose to include only cell lines for the initial investigation with this array. Tumour samples from SCLC are infrequently available for research purposes and the DNA obtained from tumour samples is usually of lower yield and lower quality than that obtained from cell lines. In all, 15 cell lines generated from SCLC, described in Table 1 (Phelps *et al*, 1996), were hybridised to the 1p array. Copy number alterations were detected in each of the 15 cell lines, ranging in size from 0.2 to 28 Mb. The locations and sizes of the alterations are summarised in Figure 1. Two cell lines (NCI-H1672, NCI-H2171) showed deletions only, eight (NCI-H82, NCI-H187, NCI-H289, NCI-H378, NCI-H889, NCI-H1184, NCI-H2195, NCI-H2227) showed amplifications only, and five (NCI-H526, NCI-H1607, NCI-H1963, NCI-H2141, HCC33) showed both amplifications and deletions. Three examples of array CGH profiles are shown in Figure 2, illustrating the detection of sub-Mb size copy number changes, as well as multiple distinct alterations along the 1p arm in NCI-H1672, NCI-H2141, and HCC33.

**Figure 3:**
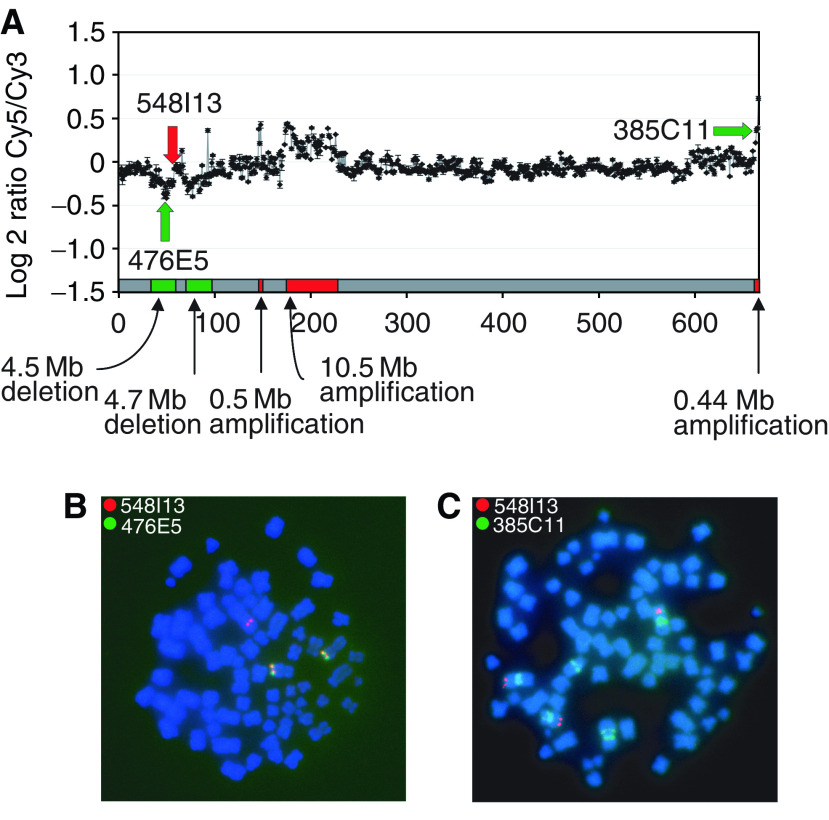
(**A**) Array CGH profile of SCLC cell line NCI-H526 demonstrating multiple segmental alterations across the 1p arm. Three BACs used in FISH analysis are indicated. (**B**) FISH analysis of 548I13 *vs* 476E6, indicating that the presence of three copies of 548I13 and two copies of 476E6. (**C**) FISH analysis of 548I13 *vs* 385C11, indicating the presence of three copies of 548I13 and six copies of 385C11. FISH experiments were conducted as described previously (Henderson *et al*, 2004).

Figure 3A shows the complex profile of cell line NCI-H526. We observe two distinct deletions at 1p36.12–p36.13 and at 1p36.21–p36.22. The area of retention between these deletions is approximately 2.5 Mb in size. The use of lower resolution techniques may have defined this region as one large deletion. Additionally, array CGH analysis was able to detect two sub-Mb alterations: a 0.5 Mb amplification at 1p35.2 and a 0.44 Mb amplification at 1p11.2–p12. Alterations of these sizes would likely have been overlooked when using a CGH array with greater than 1 Mb resolution. In order to determine if the changes in array signal intensities reflect true copy number alterations, we selected three loci for FISH experiments. The loci and their associated clones' locations are noted in Figure 3A. As seen in metaphase nuclei of NCI-H526, 548I13 shows three copies, whereas 476E5 shows two copies (Figure 3B). In a second experiment, 548I13 again shows three copies, whereas 385C11 shows six copies (Figure 3C). The normalised ratio of BAC 548I13 is at zero, even though it has three copies, as NCI-H526 has greater than a diploid number of chromosomes. These data demonstrate the ability of array CGH to distinguish loci that differ in copy number by 0.67 times (3 *vs* 2) and two times (3 *vs* 6).

Two regions of sub-Mb amplification were common among 45–50% of the cell lines. Region 1 is a 580 kb amplification at 1p34.2–p34.3, and Region 2 is a 270 kb amplification at 1p11.2, as displayed in Figure 1.

The Region 1 amplification occurs in seven of the 15 cell lines and contains BACs 314P18, 428O4, 204L3, and 115D7. Notably, in four of the seven cell lines (NCI-H378, NCI-H889, NCI-H1963, HCC33), the amplified region shows at least one BAC with a log 2 ratio greater than 2.5, corresponding to a greater than five-fold increase in signal from normal, and indicating high-level amplification. The region contains 12 genes, listed in Table 2. Conspicuously, *MYCL1*, a gene first described in SCLC (Nau *et al*, 1985) is present in this region. Of the 15 cell lines described here, all but HCC33 were previously analysed for *MYC* family amplifications using Southern blot analysis (Johnson *et al*, 1996). NCI-H378 and NCI-H889 were described as having *MYCL1* amplification, and in the current study, these amplification were also detected. We describe NCI-H526, NCI-H1184, NCI-H2141, and NCI-H1963 as having amplifications in the *MYCL1* region; however, the Southern analysis did not find *MYCL1* gene amplification in these cell lines. A possible explanation is that these cell lines do contain the amplification but they did not meet the four-fold signal increase cutoff necessary for the determination of an amplification in the Southern blot analysis. All other cell lines in which *MYCL1* amplifications were not seen by Johnson *et al* were also not seen amplified in the present study.

Previous conventional CGH studies of SCLC have revealed amplification in the *MYCL1* region. In SCLC cell lines, Levin *et al* (1994) detected amplification of chromosome bands 1p22–32 in nine of 18 samples (Levin *et al*, 1994). (*MYCL1* was initially mapped to 1p32 (Nau *et al*, 1985), then reassigned to 1p34.3 (Speleman *et al*, 1996)). Ried *et al* (1994) studied primary SCLC tumours and observed amplification of 1p32 in two of 13 cases (Ried *et al*, 1994). While these observations do implicate the *MYCL1* locus, the regions identified are approximately 40 and 10 Mb in size, respectively, and contain hundreds of genes, making the identification of the specific genes affected by the amplification virtually impossible. Using high-resolution array CGH, we have defined the precise boundaries of the amplification in each cell line, with NCI-H2141 and HCC33 having amplified regions of less than 600 kb in size. We also determined that the minimal region of alteration at 1p34.2–p34.3 is 580 kb in size and contains 11 genes in addition to *MYCL1*. This demonstrates the resolving power of array CGH to identify very small alterations and to delineate minimal regions of alteration. Previous studies that focused only on the presence and not the extent of *MYCL1* amplification may have overlooked the potential involvement of neighbouring genes.

The Region 2 amplification is a 270 kb amplification occurring in eight cell lines and contains BACs 385C11, 498H23, and 114O18. The 114O18 is the last BAC mapped to the centromeric end of the 1p arm. Two genes, *ADAM30* and *Notch2*, reside in this region. *ADAM30* is a member of the family of membrane proteins that contain a disintegrin and metalloprotease domain and its normal expression has only been detected in the testis (Cerretti *et al*, 1999). While *ADAM30* itself has not been implicated in cancer, a number of other *ADAM* genes have, such as *ADAM9* in breast cancer (O'Shea *et al*, 2003) and *ADAM12* in bone cancer (Tian *et al*, 2002). *Notch2* and the three other genes in its family, *Notch1*, *Notch3*, and *Notch4*, code for evolutionarily conserved Type 1 transmembrane receptor proteins (Artavanis-Tsakonas *et al*, 1999). While these proteins have been widely studied, investigations into their possible roles in cancer have yielded a number of studies with differing conclusions. *Notch1* was first described as being involved in a balanced translocation with *TCRβ* and a constitutively active Notch1 was oncogenic in T cells (Ellisen *et al*, 1991). Notch3 overexpression has been shown in NSCLC (Dang *et al*, 2000); however, it has also been demonstrated that expression of Notch1 and Notch2 induced cell cycle arrest in SCLC cell lines (Sriuranpong *et al*, 2001).

We searched for alterations of Regions 1 and 2 in NSCLC. In the analysis of nine NSCLC cell lines, we detected amplification of Region 1 in one sample and Region 2 in four samples (data not shown). In comparison to SCLC, the amplifications in NSCLC included larger regions and, in the case of Region 1, were at a lower level. For example, NCI-H520, a lung squamous cell carcinoma, has a 7 Mb amplification containing Region 1, and a 3.2 Mb amplification containing Region 2. The lower frequency of Region 1 amplification is in agreement with a previous observation that *MYC* family gene amplification occurs at a frequency of less than 5% in NSCLC (Bruce Johnson, personal communication), as opposed to up to 36% in SCLC (Johnson *et al*, 1996). The amplification of Region 2 was also detected in NSCLC tumours, where overexpression of *Notch2* was observed (Garnis *et al*, 2005).

**Figure 4:**
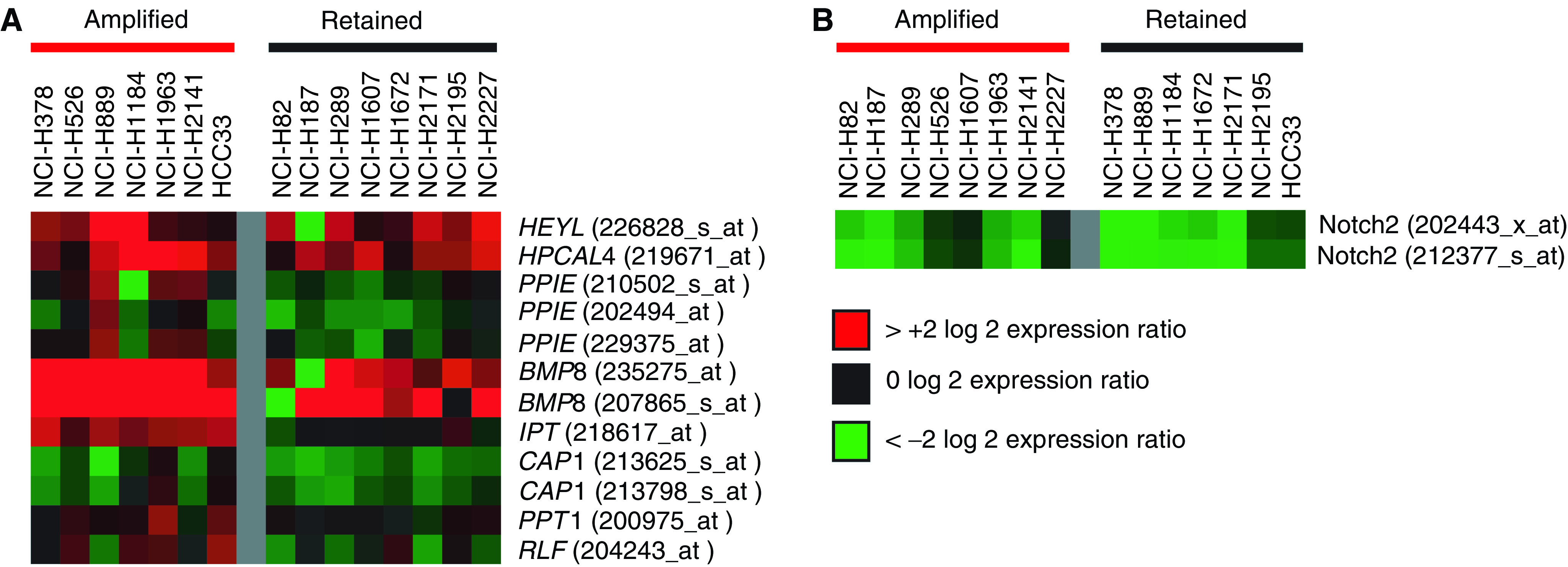
Integrating gene expression profiles with genomic alterations. (**A**) Relative expression of the genes within Region 1. (**B**) Relative expression of the genes within Region 2. Each Affymetrix probe set (probe set ID is indicated in brackets), which exhibited a detection *P*-value of less than 0.06 in >50% of samples was analysed by dividing the expression value for the gene in each experiment by the average expression value for that gene in the five normal cell lines. The resultant log 2 ratio is displayed colorimetrically according to the legend.

Gene expression analysis for each of these cell lines was obtained using Affymetrix GeneChips HG-U133A and HG-U133B. Expression levels of the genes contained in the Region 1 and Region 2 amplifications were compared between the SCLC cell lines and normal control cell lines SAEC, NHBEC, and four immortalised HBEC lines. Additionally, as there is no normal tissue that is known to be a suitable control for gene expression in SCLC, we also compared gene expression among cell lines with and without amplifications in the regions of interest. It has been previously demonstrated that the overexpression of some of the genes in a tumour can be attributed to their genomic amplification. Up to 44% of amplified genes (depending on their level of amplification) were found to be overexpressed in breast cancer cell lines (Hyman *et al*, 2002). We further compared the expression levels of these two sets of SCLC cell lines using semiquantitative RT–PCR analysis for the genes where a significant difference in expression was observed in the Affymetrix data. The results for all analyses of gene expression are detailed in Table 2 and a comparison of relative expression levels is displayed in Figure 4.

In Region 2, *ADAM30* expression was not detected on the Affymetrix array. *Notch2* was underexpressed in SCLC compared to normal cell lines. This observation is consistent with the observations of Sriuranpong *et al* (2001), where overexpression of Notch2 in SCLC induced cell cycle arrest. No difference in *Notch2* expression was observed between SCLC cells with and without the genomic amplification of Region 2 in either Affymetrix or RTPCR analyses (see Figure 4B and Table 2). It appears that the amplification of this region may not have a functional role in increasing the expression of the genes contained in it.

A number of expression differences in both comparisons were observed for the genes in Region 1. In comparing SCLC and normal cells, four genes demonstrated significant differences: *HEYL*, *HPCAL4*, *BMP8*, and *CAP1. CAP1* showed underexpression, while the other three were overexpressed. The comparison of SCLC cells with and without amplification of Region 1 revealed three genes that were overexpressed in the amplified cells: *BMP8*, *IPT*, and *RLF*. One gene, *PPIE*, was observed overexpressed in one of its three Affymetrix probes, but no difference was observed in its other two probes or the RT–PCR analysis. This may indicate a problem with that particular probe on the Affymetrix array. All other genes for which RT–PCR analysis was conducted agreed with the Affymetrix analysis (see Table 2). *BMP8* was the only gene overexpressed in both comparisons. Owing to the possibility of the comparison of expression between SCLC and normal cells being skewed by extremely high expression in the amplified samples, we compared *BMP8* expression between the normal cell lines and the nonamplified SCLC cells. *BMP8* remained overexpressed in SCLC (data not shown).

HEYL is part of a subfamily of bHLH (basic helix–loop–helix) transcription factors. These proteins control cell fate decisions such as segmentation, neurogenesis, and myogenesis (Steidl *et al*, 2000). The mouse homologue of HEYL has been shown to be a target of Notch1 signalling during development (Leimeister *et al*, 2000). As mentioned above, the Notch pathway has been found to have differing roles in cancer, depending on the tumour type.

HPCAL4 expression has only been detected in the brain. It is part of neuron-specific calcium-binding protein family; however, the specific function of HPCAL4 is not known (Kobayashi *et al*, 1998). *HPCAL4* expression was not present in the normal lung cell lines (based on the detection *P*-value), but expression was seen in the SCLC cells. This may be a result of the neuroendocrine nature of SCLC.

CAP1 is associated with actin and cofilin and allows the rapid turnover of actin filaments. This is an important function in cell motility. In yeast, CAP is a component of the Ras pathway; however, this role has not been identified in humans (Moriyama and Yahara, 2002). Differential expression of *CAP1* has not been previously detected in cancer; however, a potential role for the actin and cofilin complexes may be increasing cell motility during metastasis. A role such as this would imply overexpression of the genes involved, whereas we have observed underexpression of *CAP1*.

BMP8 is a bone morphogenetic protein, part of the TGF-*β* superfamily. These proteins play a role in aspects of mammalian development such as mesoderm determination, neural patterning, organogenesis, and skeletal patterning (DiLeone *et al*, 1997). There have been a number of studies of BMPs in cancer. BMPs were identified as potential tumour suppressor genes in myeloma (Hjertner *et al*, 2001) and prostate cancer (Brubaker *et al*, 2004). They were also shown to be overexpressed in oral squamous cell carcinoma (Jin *et al*, 2001) and *BMP7* was overexpressed in breast cancer cell lines (Hyman *et al*, 2002).

*RLF* has been shown to be in fusion with *MYCL1* in SCLC. Kim *et al* (1998) detected genomic amplification of *RLF* in four of 11 *MYCL1*-amplified SCLC cell lines they studied. Chimeric *RLF*-*MYCL1* transcripts were detected in NCI-H889, NCI-H1836, and NCI-H1994 (Kim *et al*, 1998). *RLF* contains zinc-finger motifs, and is related to the Zn-15 transcription factor. It is thought to have a general role in transcriptional regulation (Makela *et al*, 1995). The RLF-MYCL1 fusion protein has also been detected in SCLC primary tumours. Its role is thought to be the deregulation of expression of *MYCL1* (Makela *et al*, 1992). Interestingly, while *RLF* was originally identified due to fusion and overexpression with *MYCL1*, it was not the cell lines with the highest amplification in which we observed the highest expression. While NCI-H378 and NCI-H889 show the highest amplification of the region, and were the only two cell lines with previously known *MYCL1* amplification, NCI-H1184 and NCI-H1963 both express *RLF* at higher levels than NCI-H378 and NCI-H889. The observation of increased expression of *RLF*, particularly in cell lines that had not been previously studied, lends additional weight to its implication in SCLC.

The overexpression of *IPT* was the most significantly associated with amplification (see Figure 4 and Table 2). IPT catalyses the transfer of an isopentenyl group from dimethylallyl pyrophosphate to a tRNA in the biosynthesis of the tRNA cytokinin isopentenyladenosine (Golovko *et al*, 2000). Isopentenyladenine is an end product of the mevalonate pathway. Mevalonate is the precursor of isoprenoid groups that are incorporated into other end products in addition to isopentyladenine such as sterols and ubiquinone (Goldstein and Brown, 1990). HMG-CoA reductase catalyses the conversion of HMG-CoA to mevalonate. Elevated activity of this protein has been observed in a number of tumour types. The statin family of drugs are HMG-CoA reductase inhibitors that reduce levels of mevalonate and its end products. Statins have been successfully used in the treatment of hypercholesterolaemia, and have been recently demonstrated to have antiproliferative and proapoptotic effects in tumours both *in vitro* and *in vivo* (Wong *et al*, 2002). As the mevalonate pathway has been shown to play an important role in the maintenance of the malignant phenotype, *IPT*, which contributes to the production of an end product, may have a role in promoting malignancy as well. Its specific function and contribution remain to be clarified.

We have presented a chromosome arm-specific tiling resolution BAC array consisting of 642 BAC clones spanning 120 Mb of chromosome arm 1p and profiled 15 SCLC cell lines using this array. We have reliably detected the previously known *MYCL1* amplification, as well as defined a 580 kb amplification at 1p34.2–p34.3, and a novel 270 Kb Amplification at 1p11.2. This demonstrates the ability of high-resolution array CGH to identify small alterations that may have escaped detection by other means, and to detect efficiently the chromosomal location, size, and relative level of a genetic alteration. Further, we have analysed the expression of the genes contained in the amplicons, in order to identify those with a potential role in SCLC. *Notch2* and *CAP1* were underexpressed, while *HEYL*, *HPCAL4*, and *BMP8* were overexpressed in SCLC in comparison to normal cell lines. *IPT*, *BMP8*, and *RLF* were overexpressed in SCLC cells in which they were genomically amplified in comparison to SCLC cells without amplification. These data are in agreement with previous studies that broadly implicate the BMP family in cancer, and specifically implicate *RLF* in SCLC. Furthermore, we have observed increased expression of *HEYL*, *HPCAL4*, and *IPT*, for which additional investigation will be necessary to define their roles in cancer.

Genomic amplification is significant to cancer in that it can directly result in increased expression of amplified genes (Hyman *et al*, 2002). High-level amplifications have usually been seen associated with a particular oncogene such as *EGFR*, *MYC*, or *ERBB2* (Schwab, 1999). Here, we have described an amplicon containing the *MYCL1* oncogene, where additional genes are affected by the genomic amplification. This demonstrates the importance of integrating comprehensive genomic and gene expression analyses where all genes in a region can be studied. Further, we have demonstrated the benefit of a comparison among samples of the same tumour type as well as against normal tissues in order to reveal expression changes that may be functionally important.

## Figures and Tables

**Table 1:** SCLC cell lines analysed by array CGH and Affymetrix array (Phelps *et al*, 1996)

**Name**	**Morphology**	**Sex**	**Age (years)**	**Source of specimen**	**Stage at diagnosis**
NCI-H82	Variant	M	41	Pleural effusion	Extensive
NCI-H187	Classical	M	47	Pleural effusion	Extensive
NCI-H289	Variant	F	45	Pleural effusion	Extensive
NCI-H378	Classical	F	66	Lung	Extensive
NCI-H526	Variant	M	55	Bone marrow	Extensive
NCI-H889	Classical	F	69	Lymph node	Extensive
NCI-H1184	Classical	M	42	Lymph node	Limited
NCI-H1607	Classical	M	54	Lymph node	Extensive
NCI-H1672	Classical	M	58	Lung	Limited
NCI-H1963	Classical	M	56	Lung	Limited
NCI-H2141	Classical	M	58	Lymph node	Extensive
NCI-H2171	Classical	M	50	Pleural effusion	Extensive
NCI-H2195	Classical	M	67	Bone marrow	Extensive
NCI-H2227	Classical	M	54	Lung	Extensive
HCC33	Classical	M	52	NA	NA

SCLC=small-cell lung cancer; CGH=comparative genomic hybridisation; M=male; F=female; NA=not applicable.

**Table 2:** Expression analysis of genes in Region 1 and Region 2 amplifications

**Gene**	**Full name**	**Affymetrix U133 probe set**		**Affymetrix probe detectable^a^**	**Expression: SCLC *vs* normal**	**Expression: amplification *vs* retention**		
		**Affymetrix ID**	**Accession**			**Affymetrix**	**RT–PCR**	**Overall score**
*Region 1*								
*HEYL*	Hairy/enhancer- of-split related with YRPW motif like	226828_s_at	AL040198	Yes	*P*=0.00332 (up)	*P*=0.47754	*P*=0.07599	No change
		220662_s_at	NM_014571	No	—	—		
*NT5C1A*	5′-nucleotidase, cytosolic IA	224529_s_at	AY028778	No	—	—	—	
*HPCAL4*	Hippocalcin like 4	222091_at	AL136591	Yes	*P*=0.01098 (up)	*P*=0.16783	*P*=0.34716	No change
		219671_at	BE550384	No	—	—		
*PPIE*	Peptidylprolyl isomerase E	229375_at	AL526713	Yes	*P*=0.67684	*P*=0.02704 (up)^b^	*P*=0.23170	No change
		202494_at	NM_006112	Yes	*P*=0.06631	*P*=0.2317		No change
		210502_s_at	AF042386	Yes	*P*=0.84999	*P*=0.0603		
		221615_at	AF104013	No	—	—		
*BMP8*	Bone morphogenetic protein 8	207865_s_at	NM_001720	Yes	*P*=0.00837 (up)	*P*=0.01026 (up)	*P*=0.04693 (up)	Up
		235275_at	AA610122	Yes	*P*=0.00111 (up)	*P*=0.00295 (up)		
		207866_at	NM_001720	No	—	—		
*OXCT2*	3-oxoacid CoA transferase 2	220256_s_at	NM_022120	No	—	—	—	
*IPT*	TRNA isopentenyl transferase precursor	218617_at	NM_017646	Yes	*P*=0.17806	*P*=0.00016 (up)	*P*=0.02005(up)	Up
*MYCL1*	Avm oncogene homolog 1, lung carcinoma derived	Not included on array		—	—	—	—	
*FLJ14490*	Hypothetical protein FLJ14490	Not included on array		—	—	—	—	
*CAP1*	Adenylyl cyclase-associated protein 1	200625_s_at	NM_006367	Yes	*P*=0.00623 (down)	*P*=0.43326	*P*=0.38943	No change
		213798_s_at	AA806142	Yes	*P*=0.00623 (down)	*P*=0.14048		No change
*PPT1*	Palmitoyl-protein thioesterase 1	200975_at	NM_000310	Yes	*P*=0.15369	*P*=0.2317	—	No change
*RLF*	Rearranged L-myc fusion sequence	204243_at	NM_012421	Yes	*P*=0.79098	*P*=0.00699 (up)	*P*=0.03605 (up)	Up
								
*Region 2*								
*ADAM30*	A disintegrin and metalloproteinase domain 30	221446_at	NM_021794	No	—	—	—	
*Notch2*	Notch homolog 2	202445_s_at	NM_024408	No	—	—	*P*=0.06030	
		202443_x_at	AA291203	Yes	*P*=0.00004 (down)	*P*=0.16783		No change
		212377_s_at	AU158495	Yes	*P*=0.00015 (down)	*P*=0.26791		No change

SCLC=small-cell lung cancer; RT–PCR=reverse transcriptase–polymerase chain reaction.

a Affymetrix score of Present or Marginal in >50% of samples.

b RT–PCR analysis failed to confirm the expression difference detected by Affymetrix.

## References

[bib1] Artavanis-Tsakonas S, Rand MD, Lake RJ (1999) Notch signaling: cell fate control and signal integration in development. Science 284: 770–776 10221902 10.1126/science.284.5415.770

[bib2] Ashman JN, Brigham J, Cowen ME, Bahia H, Greenman J, Lind M, Cawkwell L (2002) Chromosomal alterations in small cell lung cancer revealed by multicolour fluorescence *in situ* hybridization. Int J Cancer 102: 230–236 12397641 10.1002/ijc.10704

[bib3] Brubaker KD, Corey E, Brown LG, Vessella RL (2004) Bone morphogenetic protein signaling in prostate cancer cell lines. J Cell Biochem 91: 151–160 14689587 10.1002/jcb.10679

[bib4] Buckley PG, Mantripragada KK, Benetkiewicz M, Tapia-Paez I, Diaz De Stahl T, Rosenquist M, Ali H, Jarbo C, De Bustos C, Hirvela C, Sinder Wilen B, Fransson I, Thyr C, Johnsson BI, Bruder CE, Menzel U, Hergersberg M, Mandahl N, Blennow E, Wedell A, Beare DM, Collins JE, Dunham I, Albertson D, Pinkel D, Bastian BC, Faruqi AF, Lasken RS, Ichimura K, Collins VP, Dumanski JP (2002) A full-coverage, high-resolution human chromosome 22 genomic microarray for clinical and research applications. Hum Mol Genet 11: 3221–3229 12444106 10.1093/hmg/11.25.3221

[bib5] Cerretti DP, DuBose RF, Black RA, Nelson N (1999) Isolation of two novel metalloproteinase-disintegrin (ADAM) cDNAs that show testis-specific gene expression. Biochem Biophys Res Commun 263: 810–815 10512762 10.1006/bbrc.1999.1322

[bib6] Chizhikov V, Zborovskaya I, Laktionov K, Delektorskaya V, Polotskii B, Tatosyan A, Gasparian A (2001) Two consistently deleted regions within chromosome 1p32-pter in human non-small cell lung cancer. Mol Carcinogen 30: 151–158 10.1002/mc.1023 11301475

[bib7] Dang TP, Gazdar AF, Virmani AK, Sepetavec T, Hande KR, Minna JD, Roberts JR, Carbone DP (2000) Chromosome 19 translocation, overexpression of Notch3, and human lung cancer. J Natl Cancer Inst 92: 1355–1357 10944559 10.1093/jnci/92.16.1355

[bib8] DiLeone RJ, King JA, Storm EE, Copeland NG, Jenkins NA, Kingsley DM (1997) The Bmp8 gene is expressed in developing skeletal tissue and maps near the Achondroplasia locus on mouse chromosome 4. Genomics 40: 196–198 9070944 10.1006/geno.1996.4533

[bib9] Ellisen LW, Bird J, West DC, Soreng AL, Reynolds TC, Smith SD, Sklar J (1991) TAN-1, the human homolog of the *Drosophila* notch gene, is broken by chromosomal translocations in T lymphoblastic neoplasms. Cell 66: 649–661 1831692 10.1016/0092-8674(91)90111-b

[bib10] Garnis C, Baldwin C, Zhang L, Rosin MP, Lam WL (2003) Use of complete coverage array comparative genomic hybridization to define copy number alterations on chromosome 3p in oral squamous cell carcinomas. Cancer Res 63: 8582–8585 14695166

[bib11] Garnis C, Campbell J, Davies JJ, Macaulay C, Lam S, Lam WL (2005) Involvement of multiple developmental genes on chromosome 1p in lung tumorigenesis. Hum Mol Genet 14: 475–482 15615770 10.1093/hmg/ddi043

[bib12] Garnis C, Coe BP, Ishkanian A, Zhang L, Rosin MP, Lam WL (2004a) Novel regions of amplification on 8q distinct from the MYC locus and frequently altered in oral dysplasia and cancer. Genes Chromosomes Cancer 39: 93–98 14603447 10.1002/gcc.10294

[bib13] Garnis C, Coe BP, Zhang L, Rosin MP, Lam WL (2004b) Overexpression of LRP12, a gene contained within an 8q22 amplicon identified by high-resolution array CGH analysis of oral squamous cell carcinomas. Oncogene 23: 2582–2586 14676824 10.1038/sj.onc.1207367

[bib14] Gazdar AF, Kurvari V, Virmani A, Gollahon L, Sakaguchi M, Westerfield M, Kodagoda D, Stasny V, Cunningham HT, Wistuba II, Tomlinson G, Tonk V, Ashfaq R, Leitch AM, Minna JD, Shay JW (1998) Characterization of paired tumor and non-tumor cell lines established from patients with breast cancer. Int J Cancer 78: 766–774 9833771 10.1002/(sici)1097-0215(19981209)78:6<766::aid-ijc15>3.0.co;2-l

[bib15] Girard L, Zochbauer-Muller S, Virmani AK, Gazdar AF, Minna JD (2000) Genome-wide allelotyping of lung cancer identifies new regions of allelic loss, differences between small cell lung cancer and non-small cell lung cancer, and loci clustering. Cancer Res 60: 4894–4906 10987304

[bib16] Goldstein JL, Brown MS (1990) Regulation of the mevalonate pathway. Nature 343: 425–430 1967820 10.1038/343425a0

[bib17] Golovko A, Hjalm G, Sitbon F, Nicander B (2000) Cloning of a human tRNA isopentenyl transferase. Gene 258: 85–93 11111046 10.1016/s0378-1119(00)00421-2

[bib18] Greshock J, Naylor TL, Margolin A, Diskin S, Cleaver SH, Futreal PA, deJong PJ, Zhao S, Liebman M, Weber BL (2004) 1-Mb resolution array-based comparative genomic hybridization using a BAC clone set optimized for cancer gene analysis. Genome Res 14: 179–187 14672980 10.1101/gr.1847304 PMC314295

[bib19] Henderson LJ, Okamoto I, Lestou VS, Ludkovski O, Robichaud M, Chhanabhai M, Gascoyne RD, Klasa RJ, Connors JM, Marra MA, Horsman DE, Lam WL (2004) Delineation of a minimal region of deletion at 6q16.3 in follicular lymphoma and construction of a bacterial artificial chromosome contig spanning a 6-megabase region of 6q16–q21. Genes Chromosomes Cancer 40: 60–65 15034870 10.1002/gcc.20013

[bib20] Hjertner O, Hjorth-Hansen H, Borset M, Seidel C, Waage A, Sundan A (2001) Bone morphogenetic protein-4 inhibits proliferation and induces apoptosis of multiple myeloma cells. Blood 97: 516–522 11154231 10.1182/blood.v97.2.516

[bib21] Hyman E, Kauraniemi P, Hautaniemi S, Wolf M, Mousses S, Rozenblum E, Ringner M, Sauter G, Monni O, Elkahloun A, Kallioniemi OP, Kallioniemi A (2002) Impact of DNA amplification on gene expression patterns in breast cancer. Cancer Res 62: 6240–6245 12414653

[bib22] International Human Genome Mapping Consortium (2001) A physical map of the human genome. Nature 409: 934–941 11237014 10.1038/35057157

[bib23] Ishkanian AS, Malloff CA, Watson SK, DeLeeuw RJ, Chi B, Coe BP, Snijders A, Albertson DG, Pinkel D, Marra MA, Ling V, MacAulay C, Lam WL (2004) A tiling resolution DNA microarray with complete coverage of the human genome. Nat Genet 36: 299–303 14981516 10.1038/ng1307

[bib24] Jin Y, Tipoe GL, Liong EC, Lau TY, Fung PC, Leung KM (2001) Overexpression of BMP-2/4, -5 and BMPR-IA associated with malignancy of oral epithelium. Oral Oncol 37: 225–233 11287276 10.1016/s1368-8375(00)00087-7

[bib25] Johnson BE, Russell E, Simmons AM, Phelps R, Steinberg SM, Ihde DC, Gazdar AF (1996) MYC family DNA amplification in 126 tumor cell lines from patients with small cell lung cancer. J Cell Biochem Suppl 24: 210–217 8806103 10.1002/jcb.240630516

[bib26] Kent WJ, Sugnet CW, Furey TS, Roskin KM, Pringle TH, Zahler AM, Haussler D (2002) The human genome browser at UCSC. Genome Res 12: 996–1006 12045153 10.1101/gr.229102 PMC186604

[bib27] Kim JO, Nau MM, Allikian KA, Makela TP, Alitalo K, Johnson BE, Kelley MJ (1998) Co-amplification of a novel cyclophilin-like gene (PPIE) with L-myc in small cell lung cancer cell lines. Oncogene 17: 1019–1026 9747881 10.1038/sj.onc.1202006

[bib28] Kobayashi M, Sakai E, Furuta Y, Takamatsu K (1998) Isolation of two human cDNAs, HLP3 and HLP4, homologous to the neuron-specific calcium-binding protein genes. DNA Seq 9: 171–176 10520747 10.3109/10425179809072192

[bib29] Leimeister C, Schumacher N, Steidl C, Gessler M (2000) Analysis of HEYL expression in wild-type and Notch pathway mutant mouse embryos. Mech Dev 98: 175–178 11044625 10.1016/s0925-4773(00)00459-7

[bib30] Levin NA, Brzoska P, Gupta N, Minna JD, Gray JW, Christman MF (1994) Identification of frequent novel genetic alterations in small cell lung carcinoma. Cancer Res 54: 5086–5091 7923122

[bib31] Makela TP, Hellsten E, Vesa J, Hirvonen H, Palotie A, Peltonen L, Alitalo K (1995) The rearranged L-myc fusion gene (RLF) encodes a Zn-15 related zinc finger protein. Oncogene 11: 2699–2704 8545128

[bib32] Makela TP, Shiraishi M, Borrello MG, Sekiya T, Alitalo K (1992) Rearrangement and co-amplification of L-myc and rlf in primary lung cancer. Oncogene 7: 405–409 1312699

[bib33] Marra MA, Kucaba TA, Dietrich NL, Green ED, Brownstein B, Wilson RK, McDonald KM, Hillier LW, McPherson JD, Waterston RH (1997) High throughput fingerprint analysis of large-insert clones. Genome Res 7: 1072–1084 9371743 10.1101/gr.7.11.1072 PMC310686

[bib34] Minna JD, Kurie JM, Jacks T (2003) A big step in the study of small cell lung cancer. Cancer Cell 4: 163–166 14522249 10.1016/s1535-6108(03)00221-6

[bib35] Moriyama K, Yahara I (2002) Human CAP1 is a key factor in the recycling of cofilin and actin for rapid actin turnover. J Cell Sci 115: 1591–1601 11950878 10.1242/jcs.115.8.1591

[bib36] Nau MM, Brooks BJ, Battey J, Sausville E, Gazdar AF, Kirsch IR, McBride OW, Bertness V, Hollis GF, Minna JD (1985) L-myc, a new myc-related gene amplified and expressed in human small cell lung cancer. Nature 318: 69–73 2997622 10.1038/318069a0

[bib37] Nomoto S, Haruki N, Tatematsu Y, Konishi H, Mitsudomi T, Takahashi T (2000) Frequent allelic imbalance suggests involvement of a tumor suppressor gene at 1p36 in the pathogenesis of human lung cancers. Genes Chromosomes Cancer 28: 342–346 10862041 10.1002/1098-2264(200007)28:3<342::aid-gcc13>3.0.co;2-a

[bib38] O'Shea C, McKie N, Buggy Y, Duggan C, Hill AD, McDermott E, O'Higgins N, Duffy MJ (2003) Expression of ADAM-9 mRNA and protein in human breast cancer. Int J Cancer 105: 754–761 12767059 10.1002/ijc.11161

[bib39] Osoegawa K, Mammoser AG, Wu C, Frengen E, Zeng C, Catanese JJ, de Jong PJ (2001) A bacterial artificial chromosome library for sequencing the complete human genome. Genome Res 11: 483–496 11230172 10.1101/gr.169601 PMC311044

[bib40] Phelps RM, Johnson BE, Ihde DC, Gazdar AF, Carbone DP, McClintock PR, Linnoila RI, Matthews MJ, Bunn Jr PA, Carney D, Minna JD, Mulshine JL (1996) NCI-Navy Medical Oncology Branch cell line database. J Cell Biochem Suppl 24: 32–91 8806092 10.1002/jcb.240630505

[bib41] Pinkel D, Segraves R, Sudar D, Clark S, Poole I, Kowbel D, Collins C, Kuo WL, Chen C, Zhai Y, Dairkee SH, Ljung BM, Gray JW, Albertson DG (1998) High resolution analysis of DNA copy number variation using comparative genomic hybridization to microarrays. Nat Genet 20: 207–211 9771718 10.1038/2524

[bib42] Ramirez RD, Sheridan S, Girard L, Sato M, Kim Y, Pollack J, Peyton M, Zou Y, Kurie JM, Dimaio JM, Milchgrub S, Smith AL, Souza RF, Gilbey L, Zhang X, Gandia K, Vaughan MB, Wright WE, Gazdar AF, Shay JW, Minna JD (2004) Immortalization of human bronchial epithelial cells in the absence of viral oncoproteins. Cancer Res 64: 9027–9034 15604268 10.1158/0008-5472.CAN-04-3703

[bib43] Ried T, Petersen I, Holtgreve-Grez H, Speicher MR, Schrock E, du Manoir S, Cremer T (1994) Mapping of multiple DNA gains and losses in primary small cell lung carcinomas by comparative genomic hybridization. Cancer Res 54: 1801–1806 8137295

[bib44] Schwab M (1999) Oncogene amplification in solid tumors. Semin Cancer Biol 9: 319–325 10448118 10.1006/scbi.1999.0126

[bib45] Snijders AM, Nowak N, Segraves R, Blackwood S, Brown N, Conroy J, Hamilton G, Hindle AK, Huey B, Kimura K, Law S, Myambo K, Palmer J, Ylstra B, Yue JP, Gray JW, Jain AN, Pinkel D, Albertson DG (2001) Assembly of microarrays for genome-wide measurement of DNA copy number. Nat Genet 29: 263–264 11687795 10.1038/ng754

[bib46] Speleman F, Van Camp G, Van Roy N (1996) Reassignment of MYCL1 to human chromosome 1p34.3 by fluorescence *in situ* hybridization. Cytogenet Cell Genet 72: 189–190 8978772 10.1159/000134185

[bib47] Sriuranpong V, Borges MW, Ravi RK, Arnold DR, Nelkin BD, Baylin SB, Ball DW (2001) Notch signaling induces cell cycle arrest in small cell lung cancer cells. Cancer Res 61: 3200–3205 11306509

[bib48] Steidl C, Leimeister C, Klamt B, Maier M, Nanda I, Dixon M, Clarke R, Schmid M, Gessler M (2000) Characterization of the human and mouse HEY1, HEY2, and HEYL genes: cloning, mapping, and mutation screening of a new bHLH gene family. Genomics 66: 195–203 10860664 10.1006/geno.2000.6200

[bib49] Tian BL, Wen JM, Zhang M, Xie D, Xu RB, Luo CJ (2002) The expression of ADAM12 (meltrin alpha) in human giant cell tumours of bone. Mol Pathol 55: 394–397 12456779 10.1136/mp.55.6.394 PMC1187278

[bib50] Wong WW, Dimitroulakos J, Minden MD, Penn LZ (2002) HMG-CoA reductase inhibitors and the malignant cell: the statin family of drugs as triggers of tumor-specific apoptosis. Leukemia 16: 508–519 11960327 10.1038/sj.leu.2402476

